# Thermomechanical Simulation of Orthogonal Metal Cutting with PFEM and SPH Using a Temperature-Dependent Friction Coefficient: A Comparative Study

**DOI:** 10.3390/ma16103702

**Published:** 2023-05-12

**Authors:** Juan Manuel Rodríguez Prieto, Simon Larsson, Mohamadreza Afrasiabi

**Affiliations:** 1 Escuela de Ciencias Aplicadas e Ingeniería, Universidad EAFIT, Cra 49 n 7–sur–50, Medellín 050022, Colombia; jmrodrigup@eafit.edu.co; 2 Department of Engineering Sciences and Mathematics, Solid Mechanics, Luleå University of Technology, 971 87 Luleå, Sweden; 3 Data-Driven & Computational Manufacturing Group, Inspire AG, 8005 Zürich, Switzerland

**Keywords:** metal cutting, numerical simulation, particle finite element method (PFEM), smoothed particle hydrodynamics (SPH), temperature-dependent friction

## Abstract

In this work, we apply the Particle Finite Element Method (PFEM) and Smoothed Particle Hydrodynamics (SPH) to simulate the orthogonal cutting chip formation of two workpiece materials, i.e., AISI 1045 steel and Ti6Al4V titanium alloy. A modified Johnson–Cook constitutive model is used to model the plastic behavior of the two workpiece materials. No damage or strain softening is included in the model. The friction between the workpiece and the tool is modeled following Coulomb’s law with a temperature-dependent coefficient. The accuracy of PFEM and SPH in predicting thermomechanical loads at various cutting speeds and depths against the experimental data are compared. The results show that both numerical methods can predict the rake face temperature of AISI 1045 with errors less than 34%. For Ti6Al4V, however, the temperature prediction errors are significantly higher than those of the steel alloy. Errors in force prediction were in the range of 10% to 76% for both methods, which compare very well with those reported in the literature. This investigation infers that the Ti6Al4V behavior under machining conditions is difficult to model on the cutting scale irrespective of the choice of numerical method.

## 1. Introduction

**Figure 1:**
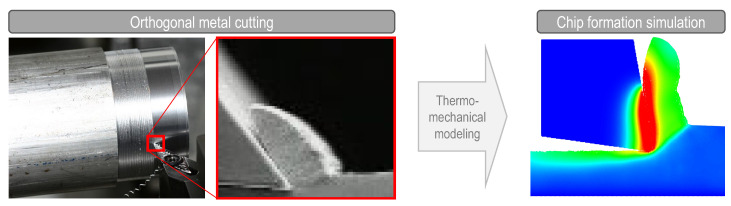
Orthogonal metal cutting process and its numerical modeling output at the scale of the tool. The left-most image created by Florian Schott, downloaded from https://commons.wikimedia.org/wiki/File:SchlichtenDrehen.jpg (accessed on 3 January 2023) under CC BY-SA 4.0.

Orthogonal cutting simulation remains a challenging task due to multiple computational and material complexities. Firstly, it involves the solution of differential equations that represent the physics of the problem in a domain that changes throughout the simulation, generating very large strains and deformations. Secondly, it needs a constitutive model that represents the behavior of the workpiece material in a wide range of strains, strain rates, and temperatures. Finally, (over) simplified friction models such as Coulomb’s law, which are mostly used in the numerical modeling framework, do not adequately represent the physics at the chip–tool interface, therefore, new friction models dependent on temperature and relative velocity are needed. The first challenge has been partially resolved with remeshing in finite element models or using completely different discretization approaches such as mesh-free or particle-based methods [1,2,3,4,5,6,7]. The second challenge is in continuous development because as the new materials appear, new constitutive models need to be developed; even so, physically based and dislocation density models have been developed that improve the prediction of the cutting forces in comparison with the Johnson–Cook constitutive model [5,8,9,10]. The last challenge appears to be less studied than the others in the numerical simulation of cutting, even though the use of better models for describing the friction behavior in metal cutting has been repeatedly concluded by many researchers in the field [11,12,13,14]. The contribution of this paper is thus directed mainly towards the third challenge in orthogonal cutting modeling. A sample of the expected results in the orthogonal cutting simulations is shown in Figure 1.

A coupled Eulerian–Lagrangian FE model as applied to predict the cutting force and chip morphology of DE 718 using a Johnson–Cook material model and a temperature dependent friction coefficient in [15], finding better predictions of cutting forces and chip shape compared to simulations with constant coefficient of friction. At the same time, a Lagrangian finite element model of metal cutting considering eight different friction coefficient models depending on the relative velocity $v_{S}$, normal contact force *F*, and temperature *T* was developed using ABAQUS/Standard in [16]. The goal was to study the influence of each friction model on the temperature distribution in the workpiece. The research found a big difference in the rising cutting temperature and in the predicted chip shapes. Moreover, a recent review of the state of the friction in metal cutting identifies that future friction models must consider thermomechanical loads [17].

In addition to the well-known Finite Element Method (FEM), particle-based methods are another spatial discretization approach frequently used for metal cutting simulations. Among the recently developed particle methods are the Particle Finite Element (PFEM) and the Smoothed Particle Finite Element (SPH) methods. Gingold et al. [18] and Lucy [19], in 1977, introduced SPH into astrophysics applications. Since the original formulation, this method has been applied to various problems, including impact [20], shock-wave simulations [21], free-surface problems [22], multi-phase flows including thermal phenomena [23], laser-based manufacturing problems, including the modeling of material removal in [24,25], pulsed-laser ablation of aluminum in [26], a ball charge and its interaction with the mill structure [27], simulation of hydraulic jump using periodic open boundaries [28], modeling dam break evolution over a wet bed [29], prediction of wear in a dumper truck body [30], and the collapse of an axisymmetric granular column of dry potassium chloride (KCl) in granular form [31].

The PFEM was initially utilized for fluid dynamics and fluid—structure interaction [32,33] problems where nonlinearities and evolving domains are present. The PFEM combines the advantages of Lagrangian particle methods with the accuracy and robustness of mesh-based methods. The PFEM discretizes the physical domain with a mesh, on which the conservation equations are solved using a Lagrangian finite element approach. In the PFEM, the mesh nodes move according to the equation of motion, behaving similar to particles and transporting their momentum together with all their physical properties. At the same time, the mesh distortion problem is overcome by generating a new mesh with a Delaunay triangulation. Unlike standard finite element schemes, the PFEM keeps the nodes of the previous mesh fixed to avoid remapping from mesh to mesh. More details about the PFEM theory are given in Section 2.3.4. PFEM is known as a numerical tool for modeling fluid and solid under big configuration changes. Examples include the application in multi-fluid flows accounting for surface tension effects [34], granular flows [35], insertion problems in geotechnical engineering [36], landslide wave generation [37], industrial forming processes [38], and fresh cement paste [39].

Within the past 15 years, several researchers have used SPH to simulate orthogonal cutting both with commercial software [40,41,42] and in-house code [4,12,43,44,45,46]. The vast majority of these papers do do not consider heat transfer between bodies or consider that the friction coefficient is constant. A friction coefficient dependent on the temperature and relative velocity was proposed in [12,47]. Previous research shows that including the dependence of friction on temperature and relative velocity improves the prediction of process forces and temperatures.

More recent than SPH, PFEM has also been used to simulate orthogonal cutting in 2D [48,49,50] and 3D [51]. Through the early developments, the method has allowed for modeling continuous [48] and segmented chips [49], including the heat transfer between the chip and the tool with a constant friction coefficient. Although previous studies have demonstrated the capability of PFEM in simulating metal cutting processes, none of them account for temperature-dependent friction coefficients. In the present work, we fill this gap by presenting a PFEM-based orthogonal cutting simulation framework featuring a temperature-dependent friction model with the coefficient values identified in [11]. The prediction accuracy of PFEM are then compared with the SPH simulation results and experimental measurements taken from the literature. The thermomechanical model created for both PFEM and SPH contains: (1) heat generation due to the plastic and frictional work, (2) heat transfer from the workpiece to the tool, (3) heat conduction in the whole system, and (4) heat loss via convection and radiation over open surfaces. No damage or fracture is taken into account.

The remainder of the manuscript is organized as follows. Section 2 presents the simulation framework for metal cutting problems with SPH and FEM. As the core of this paper, Section 3 presents the numerical results alongside experimental validation by means of force and temperature measurements. Finally, the paper closes by summarizing the key findings of this research and discussing some opportunities for further improvements, given in Section 4.

## 2. Numerical Simulation Framework

**Figure 2:**
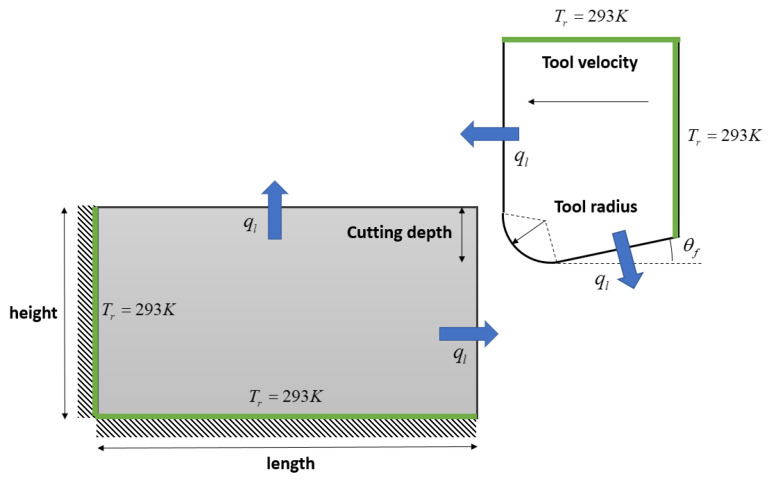
Mechanical and thermal boundary conditions for the PFEM and SPH machining simulations.

Orthogonal cutting simulations involve complex thermal, mechanical, and material phenomena. This section provides an overview about the numerical modeling of machining with PFEM and SPH without describing their derivation procedures in detail. Figure 2 illustrates the setup of an orthogonal cutting model with the mechanical and thermal boundary conditions. More details about the PFEM and SPH model of metal cutting are given in Section 2.3.5 and Section 2.4.3.

### 2.1. Balance Equations

The balance equations for a thermomechanically coupled modeling of machining in the Lagrangian representation follow the basic conservation laws (i.e., mass, momentum, and energy) and are given by:

(1) $$\begin{array}{cc} \frac{d\rho }{dt} & =-\rho \nabla \;\cdot \;\underline{v} \end{array}$$

(2) $$\begin{array}{cc} \frac{d\underline{r}}{dt} & =\underline{v} \end{array}$$

(3) $$\begin{array}{cc} \rho \frac{d\underline{v}}{dt} & =\nabla \;\cdot \;\underset{=}{\sigma }+\underline{b} \end{array}$$

(4) $$\begin{array}{cc} \rho c_{p}\frac{dT}{dt} & =k\nabla^{2}T+Q_{s} \end{array}$$

where ρ is the density, $\underline{v}$ is the velocity vector, $\underset{=}{\sigma }$ is the Cauchy stress tensor, $\underline{b}$ is the body force per unit of volume, $c_{p}$ is the specific heat capacity, *T* is the temperature, *k* is the thermal conductivity, $Q_{s}$ is the heat source due to plasticity, and $\underline{r}$ is the position vector. The heat source due to friction is included as a boundary condition at the tool–chip interface.

The term $Q_{s}$ in Equations (4) and (14) can be calculated from

(5) $$Q_{s}=\chi \sigma_{y}{\dot{\bar{\varepsilon }}}^{p}$$

where χ dimensionless parameter with range between 0 and 1, specifying the fraction of plastic work that is converted into heat, $\sigma_{y}$ is the yield stress of the material and ${\dot{\bar{\varepsilon }}}^{p}$ is the equivalent plastic strain rate.

The boundary conditions associated with Equations (2)–(4), (12) and (14) includes the heat loss that occurs via radiation and convection, the heat generation at the tool–chip interface due to friction force, imposed temperature and imposed displacement/velocity on fixed boundaries (see Figure 2). The heat loss due to convection and radiation can be expressed as:

(6) $$q_{l}=-\left[h_{c}\left(T_{s}-T_{\infty }\right)+\epsilon \sigma \left(T_{s}^{4}-T_{\infty }^{4}\right)\right]$$

in which $h_{c}$ is the heat transfer coefficient, $T_{s}$ is the temperature at the surface of the body, $T_{\infty }$ is the background temperature, ϵ is the emissivity factor, and σ is the Stefan–Boltzmann constant. Heat generated due to friction can be written as:

(7) $$q_{f}=\eta \left|f^{fric}\right|\;\cdot \;\left|v^{rel}\right|$$

where η represents the fraction of frictional work converted into heat, $\left|f^{fric}\right|$ is the magnitude of friction force, and $\left|v^{rel}\right|$ is the magnitude of the relative velocity of the chip over the tool.

In the following sections, besides an overview of material and friction modeling aspects of metal cutting, we remark on the different PDEs (but equivalent) solved by each of the numerical schemes to clarify that the displacement and pressure fields are the primary variables in PFEM, whereas the primary variable in SPH is velocity.

### 2.2. Material and Friction Modeling

This section describes the constitutive model for the workpiece as well as the friction model that will be used between the chip–tool interface.

#### 2.2.1. Flow Stress Modeling

The Johnson–Cook (JC) model [52] is used to define the plastic response of the workpiece. The von Mises stress $\sigma_{y}$ is the product of components describing strain hardening ($f_{1}$) as a function of plastic strain ${\bar{\varepsilon }}^{p}$, strain rate sensitivity ($f_{2}$) as a function of strain rate ${\dot{\bar{\varepsilon }}}^{p}$, and thermal softening ($f_{3}$) as a function of temperature *T*:

(8) $$\begin{array}{c} \sigma_{y}=\underset{f_{1}}{\underset{︸}{\left[A+B{\left({\bar{\varepsilon }}^{p}\right)}^{n}\right]}}\;\cdot \;\underset{f_{2}}{\underset{︸}{\left[1+C\ln\left(\frac{{\dot{\bar{\varepsilon }}}^{p}}{\dot{\varepsilon_{0}}}\right)\right]}}\;\cdot \;\underset{f_{3}}{\underset{︸}{\left[1-{\left(\frac{T-T_{r}}{T_{m}-T_{r}}\right)}^{m}\right]}} \end{array}$$

where *A* is the initial yield strength of the material at room temperature, *B* is the hardening modulus, *n* is the work-hardening exponent, *C* is the coefficient dependent on the strain rate, $\varepsilon_{0}$ is the reference strain rate, *m* is the thermal softening coefficient, $T_{r}$ is the room temperature, and $T_{m}$ is the melting temperature. The values of JC parameters (*A*, *B*, *C*, *m*, and  *n*) are taken from the numerical–experimental identification database of [11] and listed in Table 1.

The JC model predicts that the greater the deformations, the greater the stress. However, for steel materials starting at a strain of approximately ${\bar{\varepsilon }}^{p}=2$, the formation of new dislocations and the mutual extinction of existing dislocations remain in a state of equilibrium as deformation progresses. As a result, the yield stress remains constant for higher strain values, as was shown in [11]. To describe the behavior described above for AISI 1045 steel, the JC model of AISI 1045 will not increase for strains ${\bar{\varepsilon }}^{p}$ for ${\bar{\varepsilon }}^{p}>2$ as follows:

(9) $$\begin{array}{c} \sigma_{y}\left({\bar{\varepsilon }}^{p}>2\right)=\left[A+B{\left({\bar{\varepsilon }}^{p}=2\right)}^{n}\right]\;\cdot \;\left[1+C\ln\left(\frac{{\dot{\bar{\varepsilon }}}^{p}}{\dot{\varepsilon_{0}}}\right)\right]\;\cdot \;\left[1-{\left(\frac{T-T_{r}}{T_{m}-T_{r}}\right)}^{m}\right] \end{array}$$

The model defined above for AISI 1045 steel is referred to as the modified JC variant along this work.

#### 2.2.2. Friction Modeling

The friction force $f^{fric}$ is approximated by Coulomb’s law at the interface between the chip and the tool:

(10) $$\left|f^{fric}\right|=\mu \left(T\right)\left|f^{cont}\right|$$

where $f^{cont}$ represents the normal contact force and μ(T) is a temperature-dependent friction coefficient. The dependency of the friction coefficient on temperature follows an expression similar to the one used to model thermal softening in the Johnson–Cook flow stress model, which reads:

(11) $$\mu \left(T\right)=\mu_{0}\left[1-{\left(\frac{T-T_{r}}{T_{m}-T_{r}}\right)}^{q}\right]$$

where $\mu_{0}$ is the friction coefficient at $T=T_{r}$ and *q* is a factor that describes how fast friction decreases as temperature increases. The parameters used in the numerical simulations were obtained from the literature [11] and are presented in Table 2.

### 2.3. Particle Finite Element Modeling

This section presents an overview of machining simulation using PFEM that describes the balance equations, the spatial discretization, the temporal discretization, the meshing procedure, and the PFEM simulation setup.

#### 2.3.1. Balance Equations

In the PFEM formulation of metal cutting, the dynamic equilibrium, mass conservation, and energy balance in a Lagrangian frame consist of:

(12) $$\begin{array}{cc} \rho \frac{\partial^{2}u}{\partial t^{2}} & =\nabla \;\cdot \;\underset{=}{\sigma }+b \end{array}$$

(13) $$\begin{array}{cc} p & =\kappa \ln\left(J\right)-3\kappa \;\alpha \;\frac{\left(1-\ln(J)\right)}{J}\left(T-T_{0}\right) \end{array}$$

(14) $$\begin{array}{cc} \rho c_{p}\frac{DT}{Dt} & =k\nabla^{2}T+Q_{s} \end{array}$$

where $\underline{u}$ is the displacement vector, $\underset{=}{\sigma }$ is the Cauchy stress tensor, *b* is the body forces per unit of current volume, *p* is the pressure, κ is the Bulk modulus, α is the thermal expansion coefficient, *J* is the determinant of the deformation gradient, $T_{0}$ is the room temperature, $c_{p}$ is the specific isobaric heat capacity, *T* is the temperature *k*, the thermal conductivity and $Q_{s}$ is the heat source due to plasticity given by Equation (5). The coupling between Equations (12) and (13) is given by the decomposition of the Cauchy stress tensor $\underset{=}{\sigma }$ into isotropic and deviatoric parts:

(15) $$\underset{=}{\sigma }=p\underset{=}{I}+\underset{=}{s}$$

where $\underset{=}{I}$ is the second-order identity tensor and $\underset{=}{s}$ is the deviatoric stress tensor.

Equation (12) of the PFEM is mathematically equivalent to introduce the advection equation (Equation (2)) into the momentum equation (Equation (3)). Equation (1) is equivalent to Equation (13), the difference is due the solid approach followed in the PFEM to the modeling of the hydrostatic part of the Cauchy Stress tensor, meanwhile, SPH uses a fluid flow approach (Equation (1)). The reason to introduce the continuity equation and the decomposition of the stress tensor into its deviatoric and hydrostatic components is because we use a mixed displacement-pressure formulation with linear triangle finite elements here that handles the incompressibility constraint due to $J_{2}$ plasticity.

#### 2.3.2. PFEM Spatial Discretization of Metal Cutting Equations

The semi-discretized dynamic equilibrium and mass conservation equation are given by:

(16) $$M\ddot{\underline{u}}+{\underline{F}}^{\mathrm{int},mec}={\underline{F}}^{ext,mec}$$

(17) $$M^{p}\underline{p}-F^{p,vol}+F^{p,stab}=0$$

where *M* is the mass matrix for displacement, $\ddot{\underline{u}}$ is the nodal acceleration vector, ${\underline{F}}^{\mathrm{int},mec}$ is the vector of internal forces, ${\underline{F}}^{ext,mec}$ is the vector of external forces, $M^{p}$ is the mass matrix for pressure, $F^{p,vol}$ corresponds to the spatial discretization of the right-side term of Equation (13), and $F^{p,stab}$ is the stabilization term to overcome volumetric locking phenomena and pressure oscillations. A stabilization technique ($F^{p,stab}$ term) based on the polynomial pressure projection [53] is used in this work. More information about the spatial discretization of the dynamical equilibrium and mass conservation in PFEM is given in [5,48,49].

The thermal problem appears in the resolution of semi-discretized heat equation:

(18) $$C\dot{\underline{T}}+{\underline{F}}^{\mathrm{int},ther}={\underline{F}}^{ext,ther}$$

where *C* is the heat capacity matrix, $\underline{T}$ is the nodal temperature, ${\underline{F}}^{\mathrm{int},ther}$ is the vector of internal thermal forces, and ${\underline{F}}^{ext,ther}$ is the vector of external thermal forces. The above global force vectors are obtained as the assemblies of element vectors as it is standard in the finite elements method. In this work, the element force vectors are evaluated using Gaussian quadratures.

#### 2.3.3. Temporal Discretization in PFEM

The PFEM uses unconditionally stable implicit staggered thermomechanical algorithms, including inertial effects to integrate conservation equations presented in Section 2.3.1. It is important to briefly mention that SPH, unlike PFEM, uses an explicit time integration scheme (more information in Section 2.4.2).

In the PFEM, the thermomechanical problem is solved with an implicit staggered scheme that splits it in two sub-problems: the mechanical and the thermal part. At first, the dynamic equilibrium and mass conservation are solved at fixed temperature in a coupled way and, then, the energy balance is solved for a fixed domain. The coupling terms are included in the temperature-dependent constitutive law (see Section 2.2.1) and in the heat equation solution in the updated configuration.

Using the trapezoidal rule approximation, the time discretization of Equations (16) and (17) are written as

(19) $$\begin{array}{cc} M_{n+1}{\ddot{\underline{u}}}_{n+1}+{\underline{F}}_{n+1}^{\mathrm{int},mec} & ={\underline{F}}_{n+1}^{ext,mec} \end{array}$$

(20) $$\begin{array}{cc} M_{n+1}^{p}{\underline{p}}_{n+1}-F_{n+1}^{p,vol}+F_{n+1}^{p,stab} & =0 \end{array}$$

where $M_{n+1}$ is the mass matrix for displacement evaluated at time n+1, ${\ddot{\underline{u}}}_{n+1}$ is the nodal acceleration vector at time n+1, ${\underline{F}}_{n+1}^{\mathrm{int},mec}$ is the vector of internal forces at time n+1, ${\underline{F}}_{n+1}^{ext,mec}$ is the vector of external forces at time n+1, $M_{n+1}^{p}$ is the mass matrix for pressure at time n+1, $F^{p,vol}$ corresponds to the spatial discretization of the right side term of Equation (13) at time n+1, $F_{n+1}^{p,stab}$ is the stabilization term at time n+1.

The velocity and acceleration at time n+1 reads as

(21) $$\begin{array}{cc} {\dot{\underline{u}}}_{n+1}\; & =-{\dot{\underline{u}}}^{n}+\frac{2}{\Delta t}\left({\underline{u}}_{n+1}-{\underline{u}}_{n}\right) \end{array}$$

(22) $$\begin{array}{cc} {\ddot{\underline{a}}}_{n+1} & =-{\ddot{\underline{a}}}_{n}-\frac{4}{\Delta t}{\dot{\underline{u}}}_{n}+\frac{4}{\Delta t^{2}}\left({\underline{u}}_{n+1}-{\underline{u}}_{n}\right) \end{array}$$

where $\dot{\underline{u}}$ represents the vector of nodal velocities at time n+1. Equations (19) and (20) are solved in a coupled way using a Newton–Raphson scheme [50].

The thermal equation is solved at each time step by an implicit Backward Euler scheme numerical scheme as follows:

(23) $$C\left(\frac{{\underline{T}}_{n+1}-{\underline{T}}_{n}}{\Delta t}\right)+{\underline{F}}_{n+1}^{int,therm}={\underline{F}}_{n+1}^{ext,therm}$$

where ${\underline{T}}_{n+1}$ is the nodal temperature at time n+1, ${\underline{T}}_{n}$ is the nodal temperature at time *n*, Δt is the time interval, ${\underline{F}}_{n+1}^{int,therm}$ is the vector of internal thermal forces evaluated at time n+1, and $v_{n+1}^{ext,therm}$ is the vector of external thermal forces evaluated at time n+1. The above equation is solved with the Newton–Raphson method. Further derivation and theoretical details about the Newton–Raphson method applied to the solution of the thermal problem can be found in [50].

#### 2.3.4. PFEM Meshing

**Figure 3:**
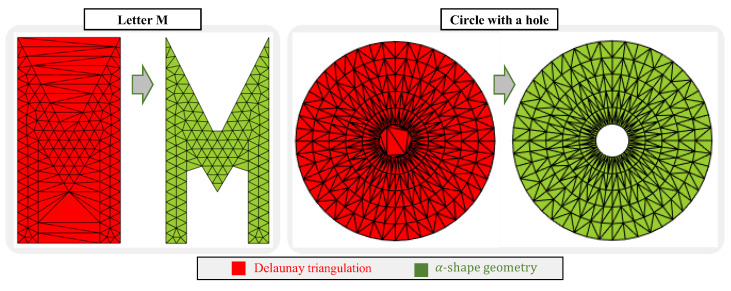
Generation of Delaunay triangulation using particles as nodes and identification of the external/internal boundary using the α-shape algorithm for two different geometries.

**Figure 4:**
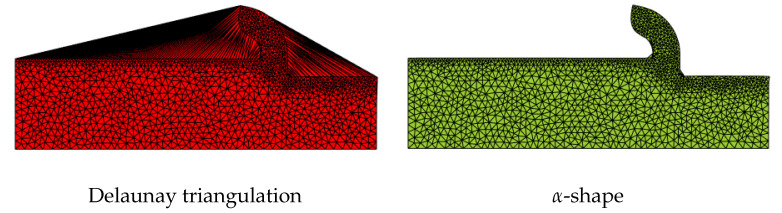
The α-shape method applied to a triangulated metal cutting geometry.

For completeness, the basics steps of the PFEM modeling framework are outlined (Algorithm 1). Given a set of particles that represent the workpiece at the beginning of the simulation.
**Algorithm 1:** Particle Finite Element Method Algorithm in Metal Cutting Simulations.step←1**while** step≤nstep **do**1.    Generate a Delaunay triangulation using the particles as nodes.2.    Identify the external and internal boundaries of the computational domain using the alpha-shape (see Figure 3 and Figure 4).3.    Implicitly solve the Lagrangian form of the mechanical and mass problem.4.    Update particles position, velocity, acceleration, pressure, and temperature.5.    Set tool incremental displacement = tool incremental displacement + time step tool displacement.    **if** remeshing is needed **then**        Go to step 1.    **else**        Go to step 3.    **end if**    nstep←nstep+1**end while**

The Delaunay triangulation of a set of points that represent a piece is shown in Figure 3, and the identification of the external/internal boundary using the alpha-shape is shown in Figure 3. It is important to remark that sometimes the alpha-shape algorithm does not match perfectly the real internal and external boundaries, as it is shown in Figure 3, where the α shape method cannot perfectly recover the shape of the letter M. Some improvements to the predicted shape of the body can be obtained by changing the value of the alpha parameter as it is shown, for example, in [54]. However, if α is too large, some distorted or really large elements can be incorporated into the mesh. On the other hand, if α is too small, many elements are removed creating nonphysical holes. Figure 3 shows an example where the choice of α can perfectly predict the point-set geometry. The α applied to the prediction of the chip shape in the numerical modeling of metal cutting processes is shown in Figure 4. Figure 4 shows that the α shape is able to predict the chip shape with high accuracy.

#### 2.3.5. PFEM Simulation Setup

**Figure 5:**
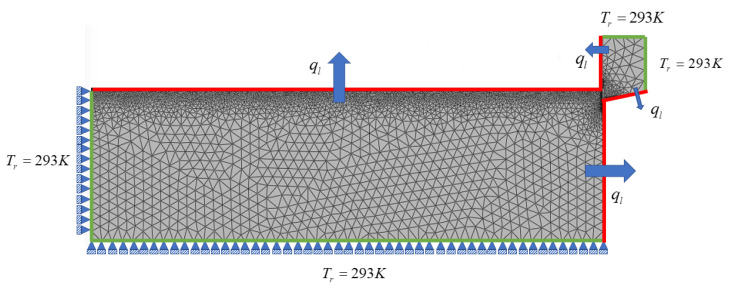
Configuration of the thermomechanical PFEM model: Initial geometry and boundary conditions.

Two-dimensional simulations of orthogonal cutting of Ti6Al4V titanium and AISI 1045 steel alloys using PFEM were carried out with the material properties, friction parameters, and process parameters given in Table A1, Table A2, and Table A3. Within the PFEM modeling framework, the workpiece was modeled as a thermo elastic–plastic body, whereby the tool is modeled as an elastic body with a cutting radius of *r* = 10 μm. Figure 5 illustrates the initial setup and boundary conditions. A constant temperature of T=293 K was imposed on the fixed surfaces (see Figure 5) as the Dirichlet boundary condition. Further, a Neumann boundary condition was imposed on the free surfaces accounting for convection and radiation heat losses (see Figure 2). The PFEM simulation runs until the tool has reached 2/3 of the workpiece length. This choice of the cutting length is made to ensure that the results are not affected by the fixed boundary condition (see the left boundary of the workpiece in Figure 2). Moreover, with the previous cutting length in all the numerical simulations a steady-state value of cutting forces is reached.

With respect to the piece geometry, the total height was chosen to be 3 times the cutting depth, and the length of the piece was set to be 3 mm for all the numerical simulations. For the discretization of the workpiece, a graded distance of particles was chosen (see Figure 5), which provides smaller distance between particles in areas with high gradients of the internal variables (typically the primary and the secondary shear zone) and where the curvature of the surface change along the simulation in order to increase the accuracy of the results. A minimum of 10, 15, and 20 particles were used along the uncut chip thickness for the cases in which $t_{u}$ = 0.1, 0.15, and 0.20 mm, respectively. The grading distance between particles used in the PFEM offers an advantage compared to other particle methods where the distance between particles is more or less constant along the simulation. Further, the same distance between nodes near the tool tip size was used to mesh the tool using linear triangle finite elements for solving displacement, elastic stresses, and temperatures. The numerical models was solved using a in-house Matlab PFEM code developed by the authors. Preliminary version of the PFEM software have been reported in [48,50,55].

### 2.4. Smoothed Particle Hydrodynamics Modeling

The Smoothed Particle Hydrodynamics (SPH) method, introduced in 1977 by Monaghan and Gingold [18] and Lucy [19], is a mesh-free Lagrangian discretization technique suitable for simulating solid and fluid flows. In SPH, the computational domain is discretized only by a set of points called particles without using a background mesh. In this section, the SPH approximations required for cutting simulations are described in brief.

#### 2.4.1. SPH Spatial Discretization of Metal Cutting Equations

The SPH method approximates a field quantity *f* using a smoothed interpolation over the particles. This allows for a particle-based approximation of the partial differential equations and is well suited for large deformations problems such as metal cutting. Using the kernel approximation and subsequent particle approximation, the basic function interpolation over the bounded domain Ω can be converted into a finite, discretized form:

(24) $$\langle f_{i}\rangle =\int_{\Omega }f\left(x^{\prime }\right)\delta \left(x-x^{\prime }\right)dx^{\prime }\approx \underset{\mathrm{kernel}\;\mathrm{approx}.}{\underset{︸}{\int_{\Omega }f\left(x^{\prime }\right)W\left(x-x^{\prime },h\right)dx^{\prime }}}\approx \underset{\mathrm{particle}\;\mathrm{approx}.}{\underset{︸}{\sum_{j=1}^{N}f_{j}W_{ij}V_{j}}}$$

where δ represents the Dirac delta function, $W(x-x^{\prime },h)$ is the SPH kernel function (denoted as $W_{ij}=W\left(x_{i}-x_{j},h\right)$) with a smoothing length *h*, *N* is the total number of neighboring particles within the support domain of particle *i*, and $V_{j}=m_{j}/\rho_{j}$ is the integration weight.

As a Lagrangian mesh-free method particularly suitable for large deformations and large strains problems, SPH is used alongside PFEM for chip formation simulations. Previous studies such as [4,40,46] have proven the efficiency of SPH in machining modeling and also shown its potential for parallel computing [12]. A brief overview of the fundamental equations required for solving Equations (1) and (2) with SPH follows.

According to [56,57], the SPH approximates the gradient ∇f and the Laplacian $\nabla^{2}f$ for a particle *i* in the following way:

(25) $$\begin{array}{cc} \langle \nabla f_{i}\rangle & \approx \rho_{i}\;\sum_{j}\left(\frac{f_{i}}{\rho_{i}^{2}}+\frac{f_{j}}{\rho_{j}^{2}}\right)\nabla W_{ij}\;m_{j} \end{array}$$

(26) $$\begin{array}{cc} \langle \nabla^{2}f_{i}\rangle & \approx \sum_{j}2\left[\left(\frac{f_{ij}}{|r_{ij}|}\right){\underline{e}}_{ij}\;\cdot \;\nabla W_{ij}\right]V_{j} \end{array}$$

where $W_{ij}=W_{h}\left({\underline{r}}_{i}-{\underline{r}}_{j},h\right)$ is a kernel function with *h* as a smoothing length and ${\underline{e}}_{ij}={\underline{r}}_{ij}/\left|r_{ij}\right|$ is a unitary vector. At the same time, the notation ${\left(•\right)}_{ij}\equiv {\left(•\right)}_{j}-{\left(•\right)}_{j}$ is used for brevity. The Wendland quintic kernel function *W* is used in this paper is, which was originally developed by [58]. In [59], it was stated that previous kernel is the optimal choice for numerical stability of SPH.

The SPH approximations presented in Equations (24)–(26) are applied to the balance equations given in Section 2.1, obtaining, as a result, the semi-discretized form of the equations as:

(27) $$\begin{array}{cc} \langle \frac{d\rho_{i}}{dt}\rangle & \approx \rho_{i}\sum_{j}{\underline{v}}_{ij}\;\cdot \;\nabla W_{ij}\;V_{j} \end{array}$$

(28) $$\begin{array}{cc} \langle \frac{d{\underline{v}}_{i}}{dt}\rangle & \approx \sum_{j}\left(\frac{{\underset{=}{\sigma }}_{i}}{\rho_{i}^{2}}+\frac{{\underset{=}{\sigma }}_{j}}{\rho_{j}^{2}}+\underset{\mathrm{stabilizers}}{\underset{︸}{\Pi_{ij}\underset{=}{I}+{\underset{=}{\Lambda }}_{ij}}}\right)\;\cdot \;\nabla W_{ij}\;m_{j}+\frac{{\underline{b}}_{i}}{m_{i}} \end{array}$$

(29) $$\begin{array}{cc} \langle \frac{dT_{i}}{dt}\rangle & \approx a_{i}\sum_{j}2\left[\left(\frac{T_{ij}}{|r_{ij}|}\right){\underline{e}}_{ij}\;\cdot \;\nabla W_{ij}\right]V_{j}+S_{i} \end{array}$$

where $a_{i}=k/\left(\rho \;c_{p}\right)$ is the thermal diffusivity of particle *i*, $S_{i}=Q_{s}/\left(\rho \;c_{p}\right)$ its heat source term due to plasticity, ∏ is the artificial viscosity, and $\underset{=}{\Lambda }$ is the artificial stress tensor. The last two terms are used for numerical stability (see their original derivation in [60,61]). A deep explanation stabilizers and their tuning parameters for SPH cutting models can be found in [12,45]. The parameters used in this work are the same as used in the mentioned references.

#### 2.4.2. Temporal Discretization

Here, a second-order leapfrog is used as an explicit time integration scheme for evolving the system of SPH particles. The maximum time step in this method is calculated *a priori* using a Courant–Friedrichs–Lewy (CFL) criterion through:

(30) $$\begin{array}{c} \Delta t\leq \underset{\Delta t_{max}}{\underset{︸}{\mathrm{CFL}\times \min\left(\Delta t_{t},\Delta t_{m}\right)}} \end{array}$$

where the time step constraint of thermal $\Delta t_{t}$ and mechanical $\Delta t_{m}$ problem are included as [62,63]:

(31) $$\begin{array}{cc} \Delta t_{t} & =\frac{\rho c_{p}h^{2}}{k} \end{array}$$

(32) $$\begin{array}{cc} \Delta t_{m} & =\frac{h}{\left(c_{0}+|\underline{v}{|}_{max}\right)} \end{array}$$

in which $c_{0}$ is the sound speed, and the CFL coefficient is taken between 0.25 and 0.40.

#### 2.4.3. SPH Simulation Setup

**Figure 6:**
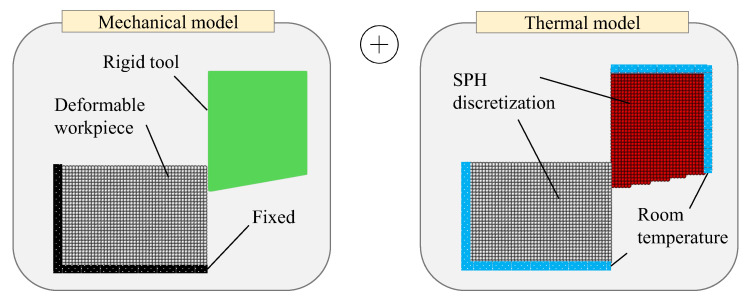
Configuration of the thermomechanical SPH model: Initial geometry and boundary conditions.

The thermomechanical SPH model of the orthogonal metal cutting test (see Figure 2) was built by discretizing the tool and workpiece with particles of uniform size. For solving the governing mechanical PDEs, we assumed the cutting tool to be a perfectly rigid body in contact with a deformable workpiece. As a result, only the workpiece was discretized by SPH particles for the mechanical parts of the simulations, while SPH interactions were inactive on the tool side. On the contrary, the governing thermal equations were solved for the entire geometry. Figure 6 illustrates the initial step of the present SPH simulation and how the thermomechanical boundary conditions are imposed on the respective surfaces.

In the SPH model setup, the workpiece dimensions were considered to be sufficiently large such that unwanted boundary effects are alleviated. To that end, we defined a 3 mm long workpiece with a height of at least 3× higher than the chip thickness in all test cases. The smoothing length factor was h=1.3Δx for all thermal and mechanical interactions. It is worth mentioning that the solver generated an identical particle spacing for discretizing the cutting tool and workpiece to ensure numerical consistency.

Furthermore, the same distance between particles was used to discretize the tool to solve the heat equation. Properties of the workpiece materials are tabulated in Table A1 and Table A2. A reduced heat capacity approach [64] was considered, in order to decrease the computing time for reaching a steady-state tool temperature. The tool is modeled as a rigid body with a radius of r = 10 μm.

In order to enable fast simulations within manageable runtimes, we utilized the GPU-accelerated version of the open-source metal cutting code **mfree-iwf**, Available on GitHub: https://github.com/iwf-inspire/mfree_iwf-ul_cut_gpu (accessed 10 January 2023). This efficient SPH solver was developed from [4,12,45,47,65,66] and can simulate 2D cutting and chip formation processes in the order of several minutes, depending on the process settings, desired resolution, and, of course, the hardware specifications. In this work, we ran all SPH parallel simulations on an nVidia™ Quadro GP100 graphics card, which is suited for scientific computing with double-precision calculations.

## 3. Results and Discussion

### 3.1. Prediction of Forces

As a first validation of the numerical simulations, forces predicted by the PFEM and SPH are compared against the experimental measurements provided by Afrasiabi et al. [11]. The comparison is made for the cutting forces $F_{c}$ and the passive forces $F_{p}$. For each material, a combination of three cutting speeds $v_{c}$ and three cutting depths $t_{u}$ was carried out. In total, for two numerical methods, two materials and nine combinations of cutting velocity/uncut chip thickness, 36 numerical simulations were performed.

**Figure 7:**
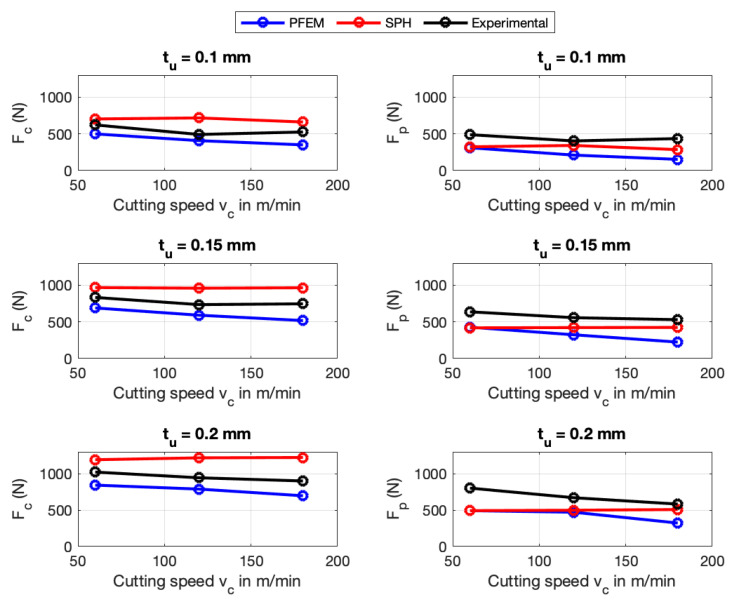
Force prediction of PFEM and SPH for AISI 1045.

**Figure 8:**
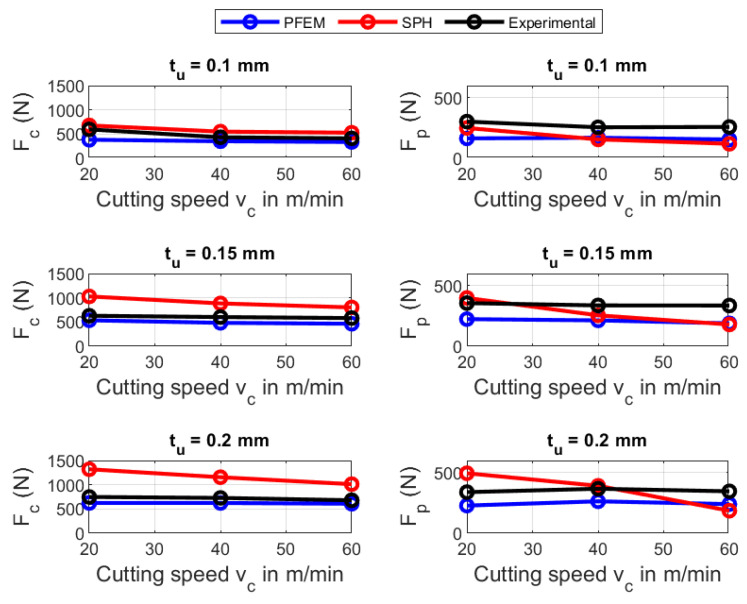
Force prediction of PFEM and SPH for Ti6Al4V.

Figure 7 and Figure 8 show the force predictions for AISI 1045 and Ti6Al4V using PFEM and SPH. The results show that, as expected, the cutting forces increases when the uncut chip thickness increases. Further, Figure 7 indicates that the predicted cutting force decrease as the cutting velocity increase. Meanwhile, for SPH and Ti6Al4V, increasing the cutting speed has a significant effect on the predicted forces in most cases (see Figure 8). At the same time, the SPH method reveals a negligible influence of the cutting speed on the predicted cutting force for AISI 1045, the same situation can be observed in the case of using PFEM for Ti6Al4V.

**Figure 9:**
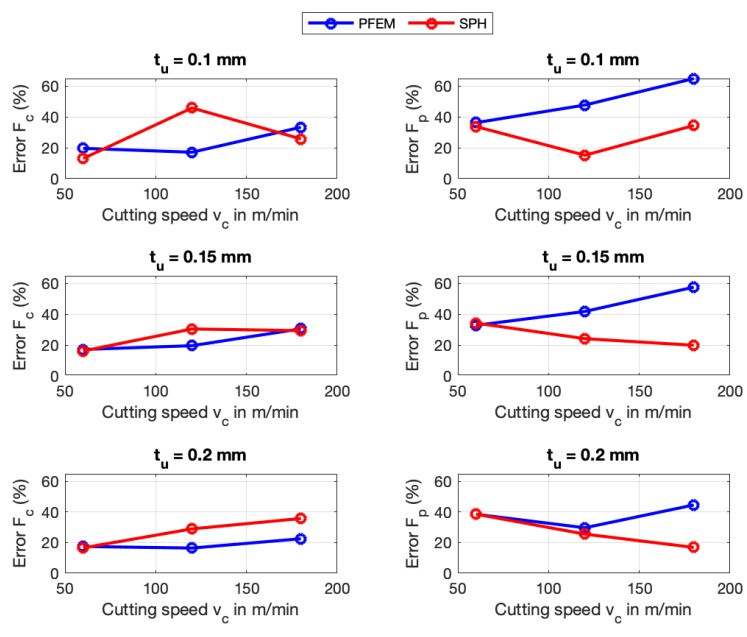
Force errors of SPH and PFEM for AISI 1045.

**Figure 10:**
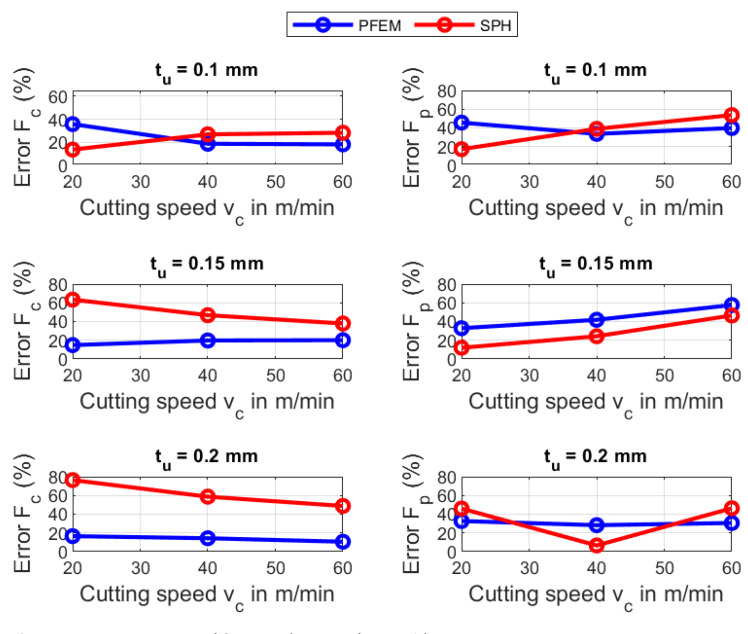
Force errors of SPH and PFEM for Ti6Al4V.

Figure 9 shows that the highest percentage error for AISI 1045 in the predicted forces using the PFEM takes places at a cutting velocity of 180 m/min and cutting depth of 0.1 mm with a relative error of 33.37% for cutting forces $F_{c}$ and 64.96% for the passive forces $F_{p}$. Figure 9 shows that the error for AISI 1045 in the predicted passives forces $F_{p}$ is around two times the error in the cutting forces $F_{c}$. The big errors in the prediction of $F_{p}$ can be attributed to the in-exactitude of friction modeling at the tool–chip interface, especially at high contact pressures and very high temperatures. Furthermore, the large error can explained by the big deviation between the calculated and the measured contact length between chip and tool. The errors in the predictions of the cutting forces $F_{c}$ and $F_{p}$ for Ti6Al4V (see Figure 10) using the PFEM are similar to the ones obtained for AISI 1045 being greater for passive forces than for cutting forces.

According to the Figure 9, for the SPH simulation of AISI 1045, the maximum error occurs at a cutting velocity of 120 m/min and a cutting depth of 0.1 mm for $F_{c}$ (45.9%) and for $F_{p}$ it occurs at a cutting speed of 60 m/min and a cutting depth of 0.2 mm with 38.6%. Figure 10 also shows the error in the cutting force $F_{c}$ and passive force $F_{p}$ using the SPH for Ti6Al4V. $F_{c}$ indicates the highest error at a cutting speed of 20 m/min and a cutting depth of 0.15 mm with 63.4%, meanwhile, $F_{p}$ reaches a highest error of 53.7%. More details about the predicted forces is given in Appendix B.

The above results show that for both materials, SPH predicts the passive forces better than PFEM. On the other hand, the PFEM outperforms SPH in terms of predicting the cutting forces. Although both numerical methods implement the same constitutive and frictional model, the differences in force predictions can be explained with the difference between the algorithms that are used to identify the boundary particles for imposing the boundary conditions in each of the numerical methods. That is, PFEM uses the α-shape [67] whereas SPH constructs a free-surface detection algorithm. In future work, a study of the influence of the free-surface detection algorithm on the predicted processes needs to be carried out.

The interpretation of the errors obtained in the prediction of the forces must take into account the strong dependence on the magnitude of the forces in the choice of the parameters of the JC model (see [55] for more details), because only changing the JC parameters can generate a significant alteration of errors. It is important to clarify that the objective of the work was to analyze the advantages and disadvantages of the numerical modeling approaches presented and not to carry out a sensitivity analysis to the input parameters (JC parameters and friction law). Follow-up developments could consider identifying the JC parameters from the cutting experiments instead of the standard material tests such as SHPB in order to improve the prediction accuracy, because the extreme strains, strain rates, and temperatures encountered in metal cutting can be reflected in the identified JC material parameters in that way. Similarly, the predictions for Ti6Al4V could be improved including some form of strain-softening [49] to obtain serrated chip formation, because the JC model alone is not able to capture chip serration [68].

### 3.2. Prediction of Thermal Loads

**Figure 11:**
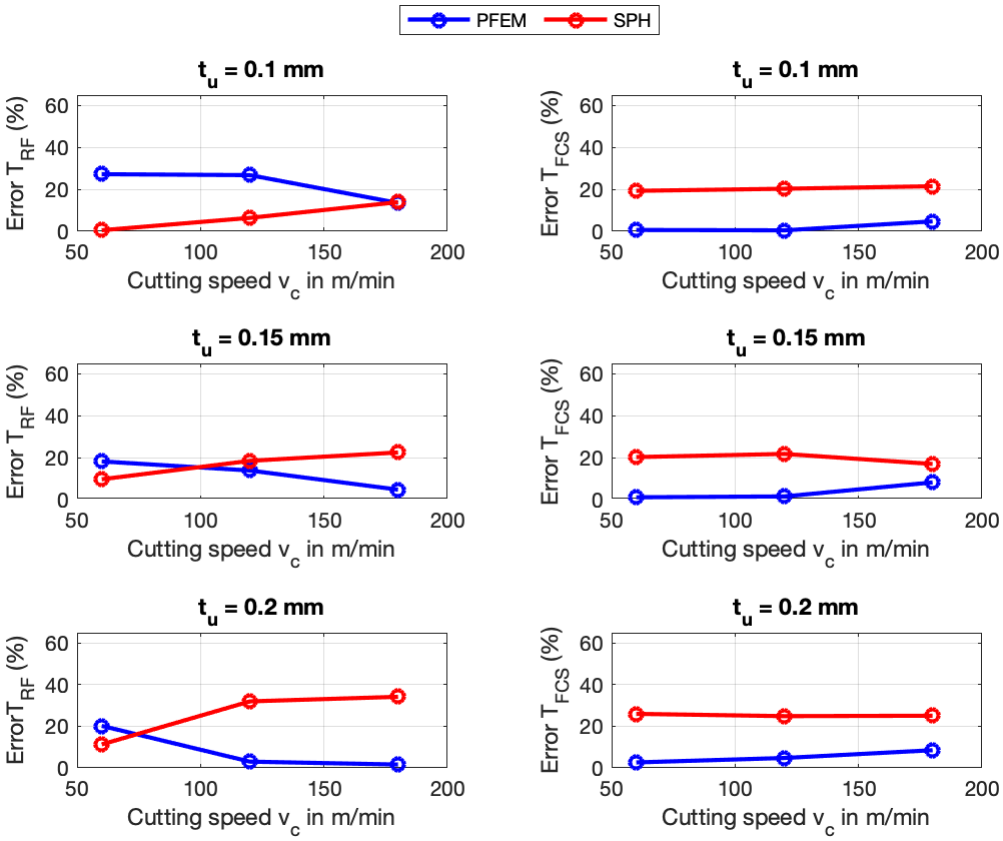
Temperature errors of SPH and PFEM for AISI 1045.

**Figure 12:**
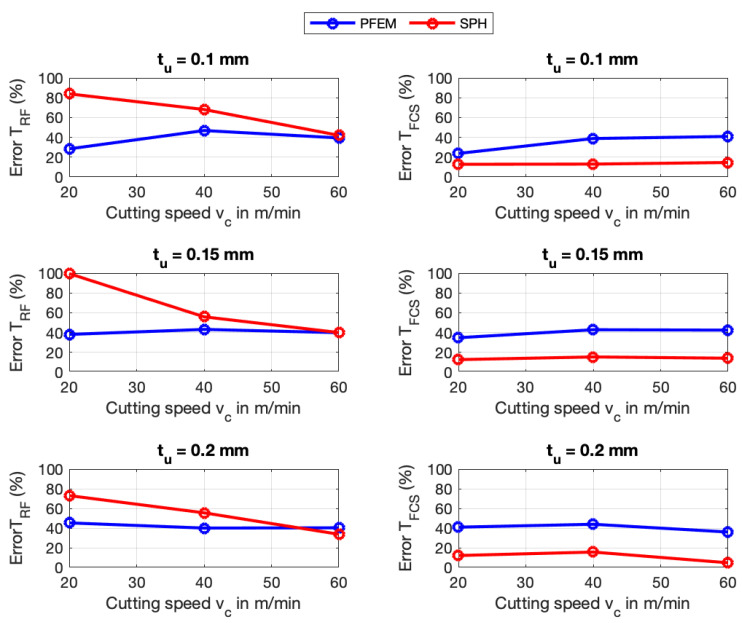
Temperature errors of SPH and PFEM for Ti6Al4V.

Numerical simulations were also validated with experimentally measured temperatures taken from [11]. In the following figures, $T_{RF}$ refers to rake face temperature and $T_{FCS}$ refers to free chip surface temperature. Figure 11 and Figure 12 show that, generally, predictions of the rake temperatures $T_{RF}$ are significantly more accurate for AISI 1045 than for Ti6Al4V in both numerical methods. It can be seen that SPH performs more accurate in predicting the free chip surface temperature $T_{FCS}$ of Ti6Al4V whereas PFEM predicts $T_{FCS}$ for AISI 1045 better than SPH. The predicted temperatures used for calculating the errors shown in the previous figures are provided in Appendix B.

Although both numerical methods implement the same constitutive and frictional model, the differences in temperature predictions are due to the fact that the methods use different algorithms to identify the particles at the boundary. A different boundary particle identification implies that the contact area between the chip and the tool is different, therefore the heat flux would be different.

The suggestions to improve the prediction of the forces made in the previous section will also help to reduce the errors in the prediction of the temperature due to the fact that coupled thermomechanical phenomena occur in the metal cutting.

### 3.3. Prediction of Chip Shapes

**Figure 13:**
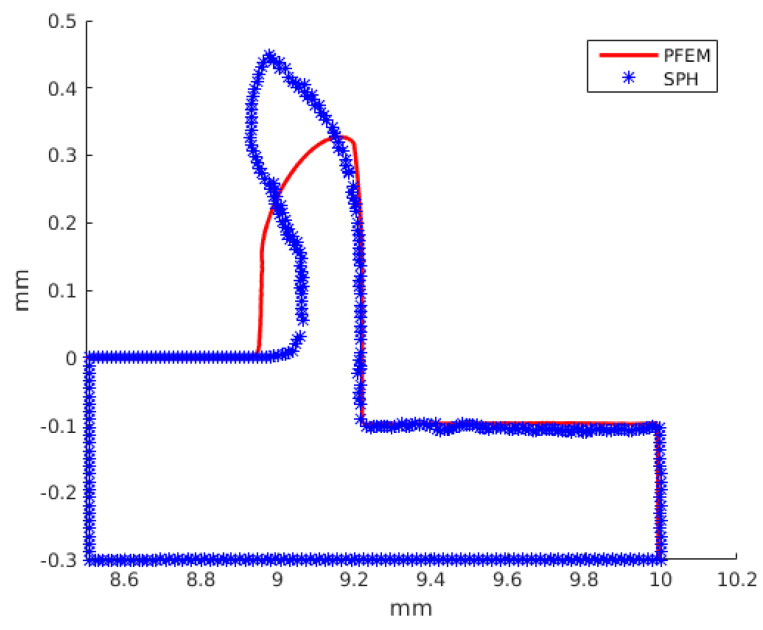
Predicted chip shapes using PFEM and SPH: Ti6Al4V, cutting speed of 40 m/s, and a uncut chip thickness of 0.1 mm.

Figure 13 shows a comparison of the predicted chips by both numerical methods for Ti6Al4V at a cutting speed of 40 m/s and a uncut chip thickness of 0.1 mm. The deformed chip thickness for the PFEM is larger compared to the SPH. The deformed chip thickness for the PFEM is larger compared to the SPH; therefore, the chip predicted by the SPH bends faster. This can be explained with the temperature predicted by the PFEM at the chip–tool interface, which, being higher than the SPH, generates higher coefficients of friction and therefore makes it more difficult for the chip to slide over the tool in PFEM simulations.

## 4. Conclusions

This paper investigated the numerical modeling of orthogonal cutting of two workpiece materials (i.e., AISI 1045 steel and Ti6Al4V) using the PFEM and the (SPH). The purpose of this work was to compare the capabilities of these two methods, which are known to be efficient for simulating large-deformation problems, in predicting forces, temperatures, and chip shapes in orthogonal cutting of two materials with different mechanical and thermal properties.

We presented the first PFEM-SPH comparison of metal cutting simulations, validated by force and temperature measurements, representing an advancement in numerical models of machining operations. For AISI 1045, the present numerical simulation framework predicted the thermal and mechanical loads of the cutting experiments with an average error below 40%. For Ti6Al4V, the prediction errors of thermomechanical loads for both numerical methods were significantly higher. This inaccuracy suggests that friction and plasticity models are far too simple to realistically describe the complex behavior at high temperatures, strains, and strain rates encountered in cutting.

The methodological difference between the two methods stems from their different time integration algorithms, i.e., implicit PFEM and explicit SPH. This has a direct impact on the calculation of contact and friction forces, thus justifying the significant discrepancy observed in some numerical cutting experiments. No concluding remark about the computational effort and runtime can be made as the two numerical methods were written in different languages (C++/CUDA vs. Matlab) and implemented on dissimilar computing platforms (GPU vs. CPU). A potential benefit of PFEM over SPH, nevertheless, appears to be the non-uniform distribution of particles enabled by the PFEM mesh structure, thereby minimizing the number of degrees of freedom required for modeling the cutting process (see Figure 5 and Figure 6).

Future development will focus on incorporating more sophisticated material models that describe the cutting process more realistically. It is also possible to speed up the PFEM calculations using GPU and other parallel computing techniques. Finally, a free-surface detection algorithm similar to SPH can be implemented into the PFEM code in order to bring the performance analysis to a more comparable state.

## Figures and Tables

**Table 1:** JC parameters for AISI 1045 and Ti6Al4V, taken from [11].

Property	AISI 1045	Ti6Al4V
*A* (MPa)	288	940
*B* (MPa)	695	965
*C*	0.0340	0.0387
*m*	1.3558	0.9835
*n*	0.2835	0.8372
$\dot{\varepsilon_{0}}$ (1/s)	0.004	0.004

**Table 2:** Parameters of temperature friction law for AISI 1045 and Ti6Al4V, taken from [11].

Material	$\mu_{0}$	*q*
AISI 1045	0.81	2.09
Ti6Al4V	0.51	5.76

## Appendix A. Thermomechanical Properties

### Appendix A.1. Workpiece

**Table A1:** Material properties of AISI 1045 and Ti6Al4V used for simulations, taken from [11].

Property	Symbol	Unit	AISI 1045	Ti6Al4V
Density	ρ	${\mathrm{kg}/m}^{3}$	7870	4430
Young’s modulus	*E*	GPa	205	110
Poisson ratio	ν	–	0.29	0.342
Heat conductivity	*k*	W/(m K)	50.7	7.44
Specific heat capacity	$c_{p}$	J/(kg K)	486	553
Melting temperature	$T_{m}$	K	1773	1877

### Appendix A.2. Tool

**Table A2:** Material properties of the carbide cutting tool, taken from [11].

Property	Symbol	Unit	Value
Density	ρ	$kg/m^{3}$	15.700
Young’s modulus	*E*	GPa	600
Poisson ratio	ν	–	0.2
Heat conductivity	*k*	W/(m K)	59
Specific heat capacity	$c_{p}$	J/(kg K)	15

### Appendix A.3. Tool–Chip Contact Zone

**Table A3:** Friction properties at the tool–chip interface, taken from [11].

Property	Symbol	Unit	Value
Reference temperature	$T_{r}$	K	293
Coeff. of plastic work into heat	*c*	–	0.9
Coeff. of frictional work into heat	*h*	–	1

## Appendix B. Predicted Forces and Temperatures

**Table A4:** Predicted forces for AISI 1045 for nine simulations obtained from the combination of three cutting velocities and three uncut chip thicknesses using PFEM.

Case	$v_{c}$ (m/min)	$t_{u}$ (mm)	γ	$\theta_{f}$	r (μm)	$F_{c}$ (N)	$F_{p}$ (N)	$F_{p}/F_{c}$
1	60	0.1	0	10	10	499.73	311.35	0.62
2		0.15				689.69	427.6	0.61
3		0.2				843.01	492.79	0.58
4	120	0.1	0	10	10	407.6	210.91	0.51
5		0.15				590.68	323.79	0.54
6		0.2				788.58	470.89	0.59
7	180	0.1	0	10	10	350.48	152.76	0.43
8		0.15				517.79	224.6	0.43
9		0.2				696.91	322.72	0.46

**Table A5:** Predicted forces for Ti6Al4V for 9 simulations obtained from the combination of 3 cutting velocities and 3 uncut chip thicknesses using PFEM.

Case	$v_{c}$ (m/min)	$t_{u}$ (mm)	γ	$\theta_{f}$	r (μm)	$F_{c}$ (N)	$F_{p}$ (N)	$F_{p}/F_{c}$
10	20	0.1	0	10	10	383.24	164.62	0.42
11		0.15				535.51	223.84	0.41
12		0.2				624.17	228.18	0.36
13	40	0.1	0	10	10	352.62	169.89	0.48
14		0.15				482.86	213.44	0.44
15		0.2				623.92	263.25	0.42
16	60	0.1	0	10	10	337	155.6	0.46
17		0.15				464.26	189.02	0.40
18		0.2				608	240.14	0.39

**Table A6:** Predicted forces for AISI 1045 for 9 simulations obtained from the combination of 3 cutting velocities and 3 uncut chip thicknesses using SPH.

Case	$v_{c}$ (m/min)	$t_{u}$ (mm)	γ	$\theta_{f}$	r (μm)	$F_{c}$ (N)	$F_{p}$ (N)	$F_{p}/F_{c}$
1	60	0.1	0	10	10	703.54	323.85	0.45
2		0.15				967.55	418.72	0.43
3		0.2				1191.91	492.56	0.41
4	120	0.1	0	10	10	717.60	342.50	0.48
5		0.15				958.99	422.12	0.44
6		0.2				1217.72	498.55	0.41
7	180	0.1	0	10	10	661.76	285.42	0.43
8		0.15				965.00	424.38	0.44
9		0.2				1222.50	507.84	0.42

**Table A7:** Predicted forces for Ti6Al4V for 9 simulations obtained from the combination of 3 cutting velocities and 3 uncut chip thicknesses using SPH.

Case	$v_{c}$ (m/min)	$t_{u}$ (mm)	γ	$\theta_{f}$	r (μm)	$F_{c}$ (N)	$F_{p}$ (N)	$F_{p}/F_{c}$
10	20	0.1	0	10	10	678.79	250.46	0.37
11		0.15				1027.53	398.65	0.39
12		0.2				1319.81	494.79	0.37
13	40	0.1	0	10	10	550.84	156.25	0.28
14		0.15				883.82	255.02	0.29
15		0.2				1155.34	391.25	0.34
16	60	0.1	0	10	10	528.64	119.94	0.23
17		0.15				800.95	179.74	0.22
18		0.2				1011.86	185.78	0.18

**Table A8:** Percent error of force prediction for AISI 1045 for 9 simulations obtained from the combination of 3 cutting velocities and 3 uncut chip thicknesses using PFEM. Mean error for $F_{c}$ = 21.57%, Mean error for $F_{p}$ = 43.81%.

Case	$F_{c}$ (N)	$F_{p}$ (N)	$F_{p}/F_{c}$
1	19.66%	36.33%	20.75%
2	17.20%	32.87%	18.92%
3	17.51%	38.55%	25.51%
4	17.15%	47.79%	36.98%
5	19.64%	41.87%	27.67%
6	16.46%	29.72%	15.87%
7	33.37%	64.96%	47.42%
8	30.59%	57.62%	38.95%
9	22.57%	44.64%	28.51%

**Table A9:** Percent error of force prediction for Ti6Al4V for 9 simulations obtained from the combination of 3 cutting velocities and 3 uncut chip thicknesses using PFEM. Mean error for $F_{c}$ = 18.77%, Mean error for $F_{p}$ = 36.47%.

Case	$F_{c}$ (N)	$F_{p}$ (N)	$F_{p}/F_{c}$
10	35.81%	45.67%	15.37%
11	14.86%	37.12%	26.15%
12	16.55%	32.69%	19.34%
13	18.75%	33.64%	18.32%
14	19.79%	36.66%	21.04%
15	14.30%	28.27%	16.30%
16	18.20%	39.92%	26.55%
17	20.09%	43.74%	29.60%
18	10.59%	30.60%	22.38%

**Table A10:** Percent error of force prediction for AISI 1045 for 9 simulations obtained from the combination of 3 cutting velocities and 3 uncut chip thicknesses using SPH. Average error $F_{c}$ = 26.92%, Average Error $F_{p}$ = 26.55%.

Case	$F_{c}$ (N)	$F_{p}$ (N)	$F_{p}/F_{c}$
1	13.1%	33.8%	41.4%
2	16.2%	34.3%	43.4%
3	16.6%	38.6%	47.3%
4	45.9%	15.2%	41.9%
5	30.5%	24.2%	41.9%
6	29.0%	25.6%	42.3%
7	25.8%	34.5%	48.0%
8	29.4%	19.9%	38.1%
9	35.8%	12.9%	35.9%

**Table A11:** Percent error of force prediction for Ti6Al4V for 9 simulations obtained from the combination of 3 cutting velocities and 3 uncut chip thicknesses using SPH. Mean error for $F_{c}$ = 44.54%, Mean error for $F_{p}$ = 32.41%.

Case	$F_{c}$ (N)	$F_{p}$ (N)	$F_{p}/F_{c}$
10	13.7%	17.3%	27.3%
11	63.4%	12.0%	31.5%
12	76.4%	46.0%	17.3%
13	26.9%	39.0%	51.9%
14	46.8%	24.3%	48.5%
15	58.7%	6.6%	32.8%
16	28.3%	53.7%	63.9%
17	37.9%	46.5%	61.2%
18	48.8%	46.3%	63.9%

**Table A12:** Predicted temperatures for AISI 1045 for 9 simulations obtained from the combination of 3 cutting velocities and 3 uncut chip thicknesses using PFEM.

Case	$v_{c}$ (m/min)	$t_{u}$ (mm)	γ	$\theta_{f}$	r (μm)	$T_{\mathrm{RF}}$ (K)	$T_{\mathrm{FCS}}$ (K)
1	60	0.1	0	10	10	850	643
2		0.15				863	646
3		0.2				880	660
4	120	0.1	0	10	10	1008	668
5		0.15				1017	667
6		0.2				1025	680
7	180	0.1	0	10	10	1050	710
8		0.15				1080	712
9		0.2				1120	714

**Table A13:** Predicted temperatures for Ti6Al4V for 9 simulations obtained from the combination of 3 cutting velocities and 3 uncut chip thicknesses using PFEM.

Case	$v_{c}$ (m/min)	$t_{u}$ (mm)	γ	$\theta_{f}$	r (μm)	$T_{\mathrm{RF}}$ (K)	$T_{\mathrm{FCS}}$ (K)
10	20	0.1	0	10	10	900	880
11		0.15				953	926
12		0.2				1120	940
13	40	0.1	0	10	10	1150	955
14		0.15				1250	950
15		0.2				1260	961
16	60	0.1	0	10	10	1280	975
17		0.15				1350	983
18		0.2				1400	990

**Table A14:** Percent error in temperature prediction for AISI 1045 for 9 simulation obtained from the combination of 3 cutting velocities and 3 uncut chip thicknesses using PFEM. Mean error for $T_{RF}$ = 14.36%, Mean error for $T_{\mathrm{FCS}}$ = 3.61%.

Case	$T_{\mathrm{RF}}$ (K)	$T_{\mathrm{FCS}}$ (K)
1	27.25%	0.62%
2	18.22%	1.10%
3	20.22%	2.64%
4	26.79%	0.45%
5	13.89%	1.52%
6	3.02%	4.78%
7	13.51%	4.72%
8	4.75%	8.21%
9	1.63%	8.51%

**Table A15:** Percent error in temperature prediction for Ti6Al4V for 9 simulations obtained from the combination of 3 cutting velocities and 3 uncut chip thicknesses using PFEM. Mean error for $T_{RF}$ = 40.16%, Mean error for $T_{FCS}$ = 38.26%.

Case	$T_{\mathrm{RF}}$ (K)	$T_{\mathrm{FCS}}$ (K)
10	28.39%	23.77%
11	38.12%	34.79%
12	45.27%	40.93%
13	46.87%	38.81%
14	43.18%	42.86%
15	40.00%	43.86%
16	39.43%	40.90%
17	39.90%	42.46%
18	40.28%	35.99%

**Table A16:** Predicted temperatures for AISI 1045 for 9 simulations obtained from the combination of 3 cutting velocities and 3 uncut chip thicknesses using SPH.

Case	$v_{c}$ (m/min)	$t_{u}$ (mm)	γ	$\theta_{f}$	r (μm)	$T_{\mathrm{RF}}$ (K)	$T_{\mathrm{FCS}}$ (K)
1	60	0.1	0	10	10	671.50	522.08
2		0.15				659.18	509.08
3		0.2				618.31	476.13
4	120	0.1	0	10	10	743.82	534.79
5		0.15				728.02	513.68
6		0.2				676.39	487.28
7	180	0.1	0	10	10	796.50	532.21
8		0.15				798.56	546.06
9		0.2				725.65	493.09

**Table A17:** Predicted temperatures for Ti6Al4V for 9 simulations obtained from the combination of 3 cutting velocities and 3 uncut chip thicknesses using SPH.

Case	$v_{c}$ (m/min)	$t_{u}$ (mm)	γ	$\theta_{f}$	r (μm)	$T_{\mathrm{RF}}$ (K)	$T_{\mathrm{FCS}}$ (K)
10	20	0.1	0	10	10	1289.11	801.86
11		0.15				1376.49	774.27
12		0.2				1334.20	747.94
13	40	0.1	0	10	10	1316.03	777.58
14		0.15				1361.25	767.15
15		0.2				1399.15	772.01
16	60	0.1	0	10	10	1303.87	792.86
17		0.15				1350.74	787.08
18		0.2				1334.11	762.14

**Table A18:** Percent error in temperature prediction for AISI 1045 for 9 simulations obtained from the combination of 3 cutting velocities and 3 uncut chip thicknesses using SPH. Mean error for $T_{RF}$ = 16.55%, Mean error for $T_{FCS}$ = 21.80%.

Case	$T_{\mathrm{RF}}$ (K)	$T_{\mathrm{FCS}}$ (K)
1	0.5%	19.3%
2	9.7%	20.3%
3	11.3%	26.0%
4	6.4%	20.3%
5	18.5%	21.8%
6	32.0%	24.9%
7	13.9%	21.5%
8	22.5%	17.0%
9	34.2%	25.1%

**Table A19:** Percent error in temperature prediction for Ti6Al4V for 9 simulations obtained from the combination of 3 cutting velocities and 3 uncut chip thicknesses using SPH. Mean error for $T_{RF}$ = 61.28%, Mean error for $T_{FCS}$ = 12.77%.

Case	$T_{\mathrm{RF}}$ (K)	$T_{\mathrm{FCS}}$ (K)
10	83.9%	12.8%
11	99.5%	12.7%
12	73.0%	12.1%
13	68.1%	13.0%
14	55.9%	15.4%
15	55.5%	15.6%
16	42.0%	14.6%
17	40.0%	14.1%
18	33.7%	4.7%
